# Genomic epidemiology of *Mycobacterium avium* subsp. *paratuberculosis* isolates from Canadian dairy herds provides evidence for multiple infection events

**DOI:** 10.3389/fgene.2023.1043598

**Published:** 2023-02-02

**Authors:** Alexander Byrne, Séverine Ollier, Kapil Tahlan, Franck Biet, Nathalie Bissonnette

**Affiliations:** ^1^ Department of Biology, Memorial University of Newfoundland, St. John’s, NL, Canada; ^2^ Sherbrooke Research and Development Centre, Agriculture and Agri-Food Canada, Sherbrooke, QC, Canada; ^3^ INRAE, ISP, Université de Tours, Nouzilly, France

**Keywords:** mycobacterium avium subsp. paratuberculosis, whole genome sequencing (WGS), molecular epidemiology, phylogenetic SNP based analysis, strain typing, MIRU-VNTR analysis, MLSSR typing, John’s disease

## Abstract

*Mycobacterium avium* subsp. *paratuberculosis* (MAP) is the pathogen responsible for paratuberculosis or Johne’s Disease (JD) in ruminants, which is responsible for substantial economic losses worldwide. MAP transmission primarily occurs through the fecal-oral route, and the introduction of an MAP infected animal into a herd is an important transmission route. In the current study, we characterized MAP isolates from 67 cows identified in 20 herds from the provinces of Quebec and Ontario, Canada. Whole genome sequencing (WGS) was performed and an average genome coverage (relative to K-10) of ∼14.9 fold was achieved. The total number of SNPs present in each isolate varied from 51 to 132 and differed significantly between herds. Isolates with the highest genetic variability were generally present in herds from Quebec. The isolates were broadly separated into two main clades and this distinction was not influenced by the province from which they originated. Analysis of 8 MIRU-VNTR loci and 11 SSR loci was performed on the 67 isolates from the 20 dairy herds and publicly available references, notably major genetic lineages and six isolates from the province of Newfoundland and Labrador. All 67 field isolates were phylogenetically classified as Type II (C-type) and according to MIRU-VNTR, the predominant type was INMV 2 (76.1%) among four distinct patterns. Multilocus SSR typing identified 49 distinct INMV SSR patterns. The discriminatory index of the multilocus SSR typing was 0.9846, which was much higher than MIRU-VNTR typing (0.3740). Although multilocus SSR analysis provides good discriminatory power, the resolution was not informative enough to determine inter-herd transmission. In select cases, SNP-based analysis was the only approach able to document disease transmission between herds, further validated by animal movement data. The presence of SNPs in several virulence genes, notably for PE, PPE, mce and mmpL, is expected to explain differential antigenic or pathogenetic host responses. SNP-based studies will provide insight into how MAP genetic variation may impact host-pathogen interactions. Our study highlights the informative power of WGS which is now recommended for epidemiological studies and to document mixed genotypes infections.

## 1 Introduction

*Mycobacterium avium* subsp. *paratuberculosis* (MAP) is the pathogen responsible for paratuberculosis or Johne’s Disease (JD) in ruminants worldwide. The symptoms of clinical paratuberculosis are chronic diarrhea and progressive weight loss and decreased production (Whitlock and Buergelt, 1996). In Canada, the annual economic impact of JD is estimated at $28 million USD, mainly due to reduced milk production, increased cattle mortality, and the early culling of infected animals (Rasmussen et al., 2021). In addition, this infection has raised concerns, in part due to the possible link between MAP and Crohn’s disease in humans, leading to discussions related to the impact of MAP on chronic diseases and food safety (Waddell L. A. et al., 2016; Kuenstner et al., 2017; Agrawal et al., 2021; Ekundayo et al., 2022).

MAP transmission primarily occurs through the fecal-oral route, which includes contaminated teats and milk or colostrum of infected animals allowing for direct transmission from cow to calf (Sweeney et al., 1992; Whittington and Windsor, 2009). Additional sources of MAP infection include environmental contamination (Fecteau et al., 2010; Waddell L. et al., 2016; Samba-Louaka et al., 2018) and the introduction of infected animals to a herd (Mitchell et al., 2019; Perets et al., 2022). Once transmitted, JD typically progresses into four consecutive phases (Whitlock and Buergelt, 1996). The first phase of MAP infection typically occurs in young livestock (< 2 years old). Early symptoms, namely cellular immune response and fecal shedding, greatly vary among calves (Mortier et al., 2015), making early diagnosis difficult. During the second stage of the disease, adult cows with subclinical MAP infection still do not display clinical symptoms, such as diarrhea or other visible signs, but can instead show altered cellular immune responses (Stabel, 2000; Ariel et al., 2020), with unreliable results in commonly used diagnostic tests that are used at this stage (Whitlock et al., 2000; Dargatz et al., 2001; Stabel et al., 2002). Detection of MAP using fecal culture is possible, but results are unpredictable because fecal excretion of MAP during the subclinical stage is not consistent. The third stage is referred to as clinical infection because visible signs of MAP infection appear, namely weight loss, intermittent diarrhea, and occasionally increased thirst (Whitlock and Buergelt, 1996). From the silent infection period, it can take from 2 to 5 years for clinical signs to appear, at which point MAP culture and enzyme-linked immunosorbent assays (ELISAs) often lead to positive results (Tiwari et al., 2006). If infected animals are not culled at this point, as is often the case, a fourth stage referred to as advanced clinical infection develops, where animals display severe symptoms (Whitlock and Buergelt, 1996).

On-farm biosecurity measures aim to limit the spread of infection (McKenna et al., 2006). However, the introduction into a herd of new individuals presents a risk of transmission. Due to the slow growing, persisting nature of this disease, it is difficult to establish the source of infection. Molecular characterization is proving to be a powerful strategy tracking infections and could help formulate legislation that would better limit spread. MAP can be broadly classified phylogenetically into sheep-type (S-type/Type I and III) and cattle-type (C-type/Type II) strains, where the latter also comprises bison-type (B-type) isolates based on differences in the *IS1311* insertion sequence (Collins et al., 1990; Whittington et al., 2001; de Juan et al., 2005). It has been noted that MAP isolates from different lineages are not specific to a particular host; therefore, the Type I, II and III classification is preferred (Stevenson et al., 2009; Fernandez et al., 2014; Verdugo et al., 2014; Bryant et al., 2016; Szteyn et al., 2020). Examination of the MAP genome has also allowed for the development of strain typing methods based on variable repeating elements, which may vary between strains. For example, eight MAP genomic loci are used in the variable number tandem repeats of mycobacterial interspersed repetitive units (MIRU-VNTR) method for strain typing (Thibault et al., 2007). In comparison, multilocus short sequence repeats (MLSSRs) consist of homopolymeric (monomeric or multimeric) repeats, 11 of which have been used for typing different MAP isolates (Amonsin et al., 2004). Recently, single nucleotide polymorphism (SNP) analysis-based methods have allowed for the identification of unique strains within populations at much higher resolutions than was possible previously (Perets et al., 2022). MAP is a slow-growing organism (*in vitro* doubling time of 22 h–26 h), and the mutation rate is likely to be low. Different studies have reported substitution rates ranging from 0.1 to 0.3 SNP/genome/year (Stevenson, 2015; Bryant et al., 2016; Davidson et al., 2016; Perets et al., 2022).

The purpose of this study was to examine genetic variations in MAP isolated from subclinical cows shedding the bacterium in their feces. These animals were from dairy herds located in Quebec and Ontario, two major dairy producing provinces in Canada. The use of whole genome sequencing (WGS) allowed for the detection of many distinct strains with significant genetic variation within a single herd, suggesting that cattle may have become infected with MAP through independent infection events, instead of being derived from a single parent strain that originated in a single infected animal at that farm. Such events have implications for infection source tracking and farm biosecurity, and some of our major findings are described in the following sections.

## 2 Materials and methods

### 2.1 Animal selection and sample isolation

The source populations were selected from 22 Canadian herds based on reported incidence of Johne’s disease (JD) in cows during the years 2013–2017. All animal procedures in the study obtained ethical approval from the Agriculture and Agri-Food Canada Animal Ethics Committee (AAFC-545).

Fecal samples were collected using a single-use veterinary glove. A blood sample was drawn from each JD cow using a SST (Serum Separation Tubes 8.5 mL; Becton, Dickinson and Company, Franklin Lakes, NJ). Blood tubes were centrifuged at 1,200 × g at 4°C for 10 min. Both fecal and blood samples were kept at 4°C during transport to the laboratory and then aliquoted and stored at −80°C until further analysis as described previously (Fock-Chow-Tho et al., 2017). Blood was tested using the IDEXX MAP Ab test kit (IDEXX Laboratories, Inc., Westbrook, ME). The presence of MAP in feces was confirmed by qPCR using the VetMAX^™^-Gold MAP Detection Kit (Life Technologies, Corp., Austin, TX) following DNA extraction using the ZR-96 Fecal DNA Kit (Zymo Research Corp., Irvine, CA). In the current study, 555 cows from the 3,452 tested in triplicate, displayed positive results for MAP-infection using both fecal qPCR and ELISA (data not shown). A MAP isolate from a minimum of three infected cows per herd was selected (apart from two herds for which only one cow gave culture positive results). Cows from each herd were selected based on the highest levels, i.e., those samples which had the lowest cycle threshold (Ct) value during qPCR. The mean age of the 67 cows at the time of selection for MAP culture was 5.26 ± 1.60 years. The number of MAP bacteria excreted in feces was evaluated by qPCR using standard curves of the MAP K-10 strain (ATCC BAA-968D-5) made with nine serial dilutions (5 and 2 fold, alternatively) from 10 pg to 0.001 pg (2,000–0.2 genomic copies, respectively), based on a genome size of 4.83 Mb. Standard curves were used to extrapolate genome copy number in one gram of feces.

### 2.2 Fecal decontamination procedure

The double incubation method, using hexadecylpyridinium chloride (HPC) and a mixture of antibiotics, was employed to decontaminate fecal samples as described previously (Whitlock and Rosenberger, 1990; Whittington et al., 1999). Briefly, 2.0 g of feces was added to 35 mL (for high shedders) or 15 mL (for moderate and low shedders) of sterile water. Samples were vortexed and were left to sit at room temperature for 30 min to allow particles to settle. The supernatant (5 mL) was transferred to 25 mL of 1/2x brain heart infusion broth (Hardy Diagnostics, Santa Maria, CA) containing 0.9% HPC (Sigma-Aldrich, Saint-Louis, MO), and incubated at 37°C for 18 h–24 h. After incubation, the sample tubes were centrifuged at 900 x g at room temperature for 30 min and immediately after centrifugation, the supernatant was decanted and the pellet was resuspended in 1 mL of 1/2x BHI containing 100 μg/mL of vancomycin (US Pharmacopeia, Rockville, MD), 100 μg/mL of nalidixic acid and 50 μg/mL of amphotericin B (Sigma-Aldrich). The sample was incubated at 37°C for 48 h after which it transferred to a cryogenic holding tube. The sample was then used to inoculate solid and liquid cultures and an aliquot was stored at −80°C.

### 2.3 Growth of MAP cultures

To obtain single colonies, slants of Herrold’s egg yolk agar with amphotericin, nalidixic acid, vancomycin and mycobactin J (Becton, Dickinson and Company, Sparks, MD) were inoculated with up to 100 µL of a decontaminated MAP suspension (prepared as described in Section 2.2). Slants were incubated at 37°C and observed every 2–4 weeks for up to 25 weeks. While small white colonies usually appeared after 4–6 weeks of incubation, incubation was continued beyond this period to ensure that single colonies would be collected, which are more visible when they reach > 0.5 mm in diameter. For the cows whose excretion of MAP was high, because of too high a density of colonies on the agar, the resumption of a spreading on agar of a 1:10 dilution of the decontamination product made it possible to ensure the collection of an isolated strain of MAP.

For low MAP-load samples which failed to grow sufficient colonies on the solid media, an intermediate step in liquid media between decontamination and growth on agar slant was required. As previously described (Whittington et al., 2013), two 50-mL tubes containing 6 mL of the M7H9C medium were inoculated with 100 and 400 µL of the decontaminated fecal suspension and were incubated at 37°C for up to 25 weeks. As a pellet of egg yolk, which contains MAP, had formed at the bottom of the tube, the culture was lightly vortexed weekly. From the 9th week of incubation, then every 4–6 weeks, growth of MAP was monitored by the Morse staining method. Briefly, 2 µL of the pellet of egg yolk was spread on a microscope slide and was stained using the TB Fluorescent Stain Kit M (Becton, Dickinson, and Company) according to the manufacturer’s instructions. Fluorescence of acid-fast bacilli was observed using the Evos FL Auto Microscope (Thermo Fisher Scientific, Waltham, MA) with the GFP fluorescence filter. When the density of MAP (assessed visually) was sufficient, solid cultures was processed as described above. The egg yolk pellet from the M7H9C culture was passed 4–5 times through a 26G needle to dissociate as many MAP clumps as possible. Serial dilutions (1:10; 1:100; 1:1,000; 1:10,000 depending on the observed MAP density) were inoculated into Middlebrook 7H9 medium (Becton, Dickinson, and Company) supplemented with 10% Oleic-Albumin-Dextrose-Catalase enrichment (OADC, Becton, Dickinson and Company) and 0.05% Tween 80 (Sigma-Aldrich), and then 10 µL were used to inoculate Herrold’s egg yolk agar with amphotericin, nalidixic acid, vancomycin and with mycobactin J.

After 9–15 weeks of incubation, a single colony from each cow was cultured into 1.5 mL of Middlebrook 7H9 broth supplemented with 10% OADC enrichment, 2% glycerol (Wisent Inc., Saint-Jean-Baptiste, QC, Canada) and 2 mg/L of mycobactin J (Allied Monitor Inc., Fayette, MO) at 37°C with agitation. From the 7th week then every 2–4 weeks, absorbance at 600 nm was measured until it reached 0.7 (mid log phase of bacterial growth). A portion of the culture was stored at −80°C in preserving medium containing 5% Tryptic Soy Broth (Sigma-Aldrich) and 30% glycerol, after a 15-min centrifugation at 4,000 × g. The remaining portion was centrifuged for 15 min at 14,000 x g and the pellet was stored at −80°C until DNA extraction could be carried out. A single colony from each cow was selected and an axenic culture was made from this single colony in order to obtain sufficient amount of MAP (∼10^9) for DNA extraction.

### 2.4 DNA extraction and sequencing preparation

Genomic DNA was extracted using the Quick-DNA Fecal/Soil Microbe Miniprep Kit (Zymo Research Corp.) according to the manufacturer’s instructions. Homogenization of MAP pellet was performed using an Omni Bead Ruptor 24 (Omni International Inc., Kennesaw, GA), twice for 1 min at 6.0 m/s. DNA concentration was quantified using a NanoDrop One and Qubit 4 Fluorometer (Thermo Fisher Scientific). Shotgun libraries from a total of 67 DNA isolates were prepared by the Centre d’expertise et de services Génome Québec (Montreal, QC, Canada) and sequenced using 150-bp paired-end reads with Illumina NovaSeq 6,000 technology (SP flowcell). In addition to the 67 field isolates gathered from farms, 10 NCBI reference sequences were also downloaded for comparison. These publicly available genome sequences included that of MAP K-10 (GenBank accession No. NC_002944.2), Telford (No. NZ_CP033688.1), S397 (No. NZ_CP053749.1), MAPK_JB16/15 (No. NZ_CP033911.1), NL 89C (No. NZ_LGRY01000001-NZ_LGRY01000098), NL 93B (No. NZ_LGRZ 01000001-NZ_LGRZ01000090), NL 95A (No. NZ_LGSA01000001-NZ_LGSA01000094), NL 95B (No. NZ_LGSB01000001-NZ_LGSB01 000090), NL 95E (No. NZ_LGSC01000001-NZ_LGSC01000097), and NL 96E (No. NZ_LGSD01000001-NZ_LGSD01000090).

### 2.5 Processing of sequencing data

Several bioinformatic tools were used to process the sequencing reads (Supplementary Figure S1). Fastp was used to trim adapters from the raw FASTQ files and to exclude any unpaired sequence reads that may be present (Chen et al., 2018). The trimmed FASTQ files were checked with FastQC to confirm read quality (Andrews, 2010). Except for three isolates with suspected contaminants, all results were considered acceptable. Kraken2 was then used to perform taxonomic identification of the read using a custom-built database (Wood et al., 2019) using NCBI RefSeq and 142 MAP sequences from the European Nucleotide Archive (ENA) (Conde et al., 2021). KrakenTools was used to extract reads corresponding to the family Mycobactereaceae (NCBI TXID1762) (Lu, 2020). The genome assembler SPAdes v. 3.15.4 was run using six kmer sets (21, 33, 55, 77, 99, and 127) with the “isolate” setting to assemble the reads from each isolate into contigs (Bankevich et al., 2012; Prjibelski et al., 2020). RagTag was used to improve on the contig assemblies produced by SPAdes (Alonge et al., 2022). QUAST was used on both SPAdes contig/scaffold and RagTag scaffold results to identify any outstanding quality control issues (Gurevich et al., 2013). The CheckM lineage-based workflow was used on RagTag scaffold results to examine the completeness and level of contamination present within each of the assemblies (Parks et al., 2015).

### 2.6 Annotation of MAP genomes

To annotate the assemblies, Prokka was run using four different reference strain sequences (representing type I, type II, type III, and type B MAP strains) as trusted annotation files (Seemann, 2014). Prokka HMM databases were enhanced with Pfam and TIGRFam databases (Haft et al., 2001; Finn et al., 2014) to allow additional accurate protein annotations to be added. Prokka was also run for each RefSeq strain using their respective.gff files as a reference.

### 2.7 Variant analysis

Snippy was used to call variants (including SNPs, insertions, deletions, multi-nucleotide polymorphisms, and complex variations) within both the reference genomes and the FASTQ files processed by trimming and quality assessment for each isolate (Seemann, 2015). Snippy was used four times, with each run using a different reference genome corresponding to type I, II, III and B MAP strain types. The files produced by the initial Snippy analysis were examined to determine the number, type and location of variants with respect to each reference strain. For construction of core SNP phylogenies based on each type of strain, further processing using snippy_core (for identifying and collecting core SNPs), snippy_clean (to remove SNPs of poor quality and any remaining gaps), gubbins (to find and remove any regions which may be indicative of recombination) and SNP-sites (to create the final multiple sequence alignment) was performed (Croucher et al., 2015; Page et al., 2016). Variant patterns and identification of unique SNPs were compared using Geneious Prime V. 2022.1.1 (Geneious Prime 2022.1.1, 2022). Statistical analysis was performed by ANOVA (analysis of variance) with unequal variances. Comparison of the herds was done with a Tukey correction**.** For the association with herd prevalence, Spearman Correlation analysis was performed in SAS (SAS Statistical Analysis System, Release 9.4, 2002–2012. SAS Institute Inc., Cary, NC).

### 2.8 Phylogeny construction

Data on the variant analysis allowed for the construction of four core SNP phylogenies. Each core SNP tree was created with IQ-TREE which selected the optimal model for tree building (GTR + ASC) and built each tree with 1,000 bootstraps (Nguyen et al., 2015; Minh et al., 2020). Tree visualization was performed using ITOL V6 (Letunic and Bork, 2021).

### 2.9 MIRU-VNTR and MLSSR typing

Geneious Prime V. 2022.1.1 was used to extract *in silico* MLSSR results from the assemblies (Amonsin et al., 2004). If the repeat number at a MLSSR locus was unclear, the individual reads were examined to validate the number of repeats. MIRU-VNTR repeats were counted using the Tandem Repeats Finder (Benson, 1999; Thibault et al., 2007). MIRU-VNTR and MLSSR data were reported in a datasheet file containing the isolate number and herd ID (Supplementary Table S5). Importing the data files into the Phandango visualizing software, alongside the type II phylogeny and shedding data, allowed for visual representation of the *in silico* analysis (Hadfield et al., 2018). MLSSR and MIRU-VNTR patterns were compared with those found in the MAC-INMV database to compare analyzed strains with previously characterized isolates (Cochard et al., 2020). The discriminatory index (DI), as described (Hunter and Gaston, 1988), was used to calculate the discriminatory power of both the MLSSR and MIRU-VNTR loci. The DI was calculated using the following equation:

$$DI=1-\left[\frac{1}{N\left(N-1\right)}\overset{s}{\underset{j=1}{\sum }}n_{j}\left(n_{j}-1\right)\right]$$

where *N* is the total number of isolates being typed, *s* is the total number of distinct types being discriminated by the respective typing method, and *n*_*j*_ is the number of isolates belonging to the *j*th type.

The MIRU-VNTR results for the 67 field isolates were confirmed with PCR using 5 μL Green GoTaq buffer (Promega), 1.5 μL 25 mM MgCl2 (Promega), 2.5 μL 2 mM dNTPs (Promega), 0.1 μL (5 U/μL) GoTaq G2 Flexi DNA polymerase (Promega), 2.5 μL (10 μM) forward and reverse primers (Eurogentec), and 5 μL DNA (≥ 10 ng/μL). A volume of 1 μL of dimethylsulfoxide (DMSO) (Sigma D2650) and/or 5 μL of 5 M betaine (Sigma B0300) were added as indicated (Thibault et al., 2007) and the total reaction volume was completed to 25 μL with molecular grade distilled water. The PCR was carried out using a Techne TC512 thermocycler according to the following reaction protocol: an initial incubation of 5 min at 94°C, followed by 40 cycles of 30 s at 94°C, 30 s at the temperature of specific hybridization of each locus, 30 s at 72°C, and a final cycle of 7 min at 72°C. PCR reaction products were analyzed by electrophoresis on a 1.5% agarose gel for 1 h at 100 V to determine the number of repeats present at each of the eight loci.

## 3 Results and discussion

### 3.1 Animal phenotypes and MAP isolates

**FIGURE 1:**
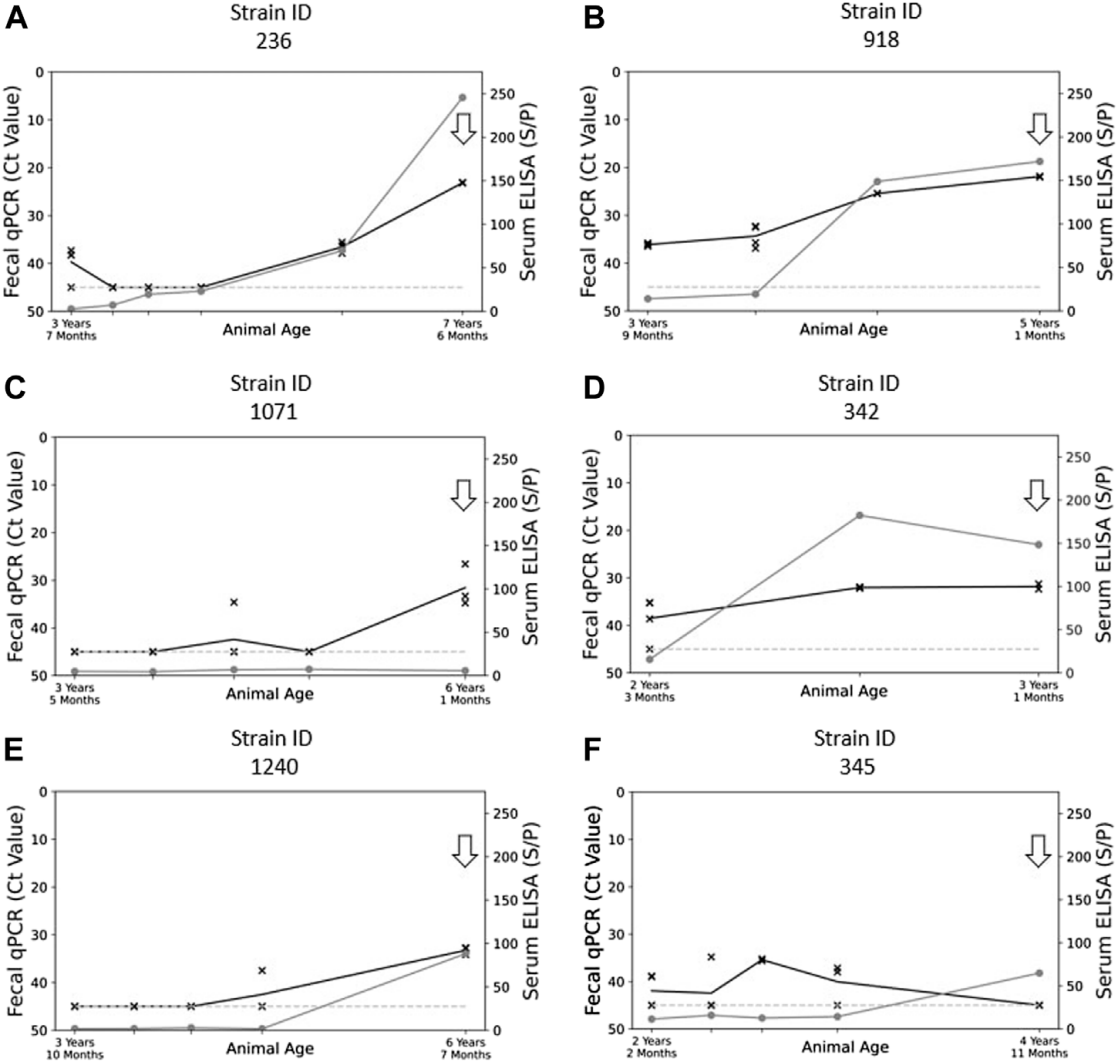
Graphical plots showing the relationship of fecal shedding of MAP (qPCR Ct value) and blood ELISA (Sample/Positive Ratio) to animal age. Panels **(A)** and **(B)** reflect the profile of phenotypes collected in “high shedding” animals (Ct < 27), panels **(C)** and **(D)** reflect the trends present in “moderately shedding” animals (27 < Ct < 33), and panels **(E)** and **(F)** reflect the trends present in “low shedding animals” (Ct > 33). The arrows present on each graph represent the timepoint in each animal that fecal samples were selected for the culture of MAP. The grey line with points represented by “O” represents the S/*p*-value of the serum ELISA at that point in time. The black line with points represented by “X” represent the Ct results (mean value) obtained from qPCR at that point in time. The dashed line along the bottom represents the minimum ELISA threshold for isolates to be suspected of being MAP positive.

Serum ELISA measures the presence of specific antibodies to MAP while fecal PCR is a direct measurement of the presence of MAP in the feces. Culture has the advantage of detecting live MAP; however, the PCR technique is quantitative enabling the evaluation of bacterial shedding levels. In a previous study, we compared different DNA extraction systems coupled with quantitative real-time PCR (qPCR) and evaluated the detection limit of MAP present in feces (Fock-Chow-Tho et al., 2017). The most efficient protocol was used to quantify fecal MAP excretion in 555 MAP-infected cows from the 3,452 cows from 22 herds that were initially tested. Samples from most animals were collected on a semi-annual basis. The specificity of fecal MAP detection is 100% because the target *ISMAP02* is specific to MAP (Stabel and Bannantine, 2005). True MAP infections were also confirmed by serum ELISA, where specificity reached 97.1%–98.6% (Jakobsen et al., 2000; Salgado et al., 2007) and fecal PCR (Fock-Chow-Tho et al., 2017). However, because of potential passive fecal shedding of MAP, the phenomenon of MAP being ingested from contaminated feed and then being detected in the feces without causing disease (Kralik et al., 2014), cultures were performed on some suspected samples to confirm MAP infections. Cows with low fecal excretion of MAP with a negative serum ELISA test were confirmed to be infected with MAP using culture (e.g., Figures 1C, f). The detection limit of qPCR is equivalent to 100–500 colony forming units (cfu)/g of feces, which corresponds to quantitative PCR values reported by the cycle threshold (Ct), of 37–38^−ΔΔCT^ (Fock-Chow-Tho et al., 2017). Similarly, high-shedding cows are known to excrete levels of MAP approaching 1 million cfu/g feces, which would correspond to Ct values < 27 according to the extrapolation made from our standard curves (Supplementary Figure S2) that are also similar to our previous observations (Fock-Chow-Tho et al., 2017).

To obtain MAP isolates from each herds, up to six cows with the highest shedding levels per herd, as detected by qPCR, were prioritized. Examination of fecal qPCR data of these cattle, with multiple samples recorded over a 3–5 years’ period in most cases, assigned the animals to three MAP shedding categories: low, moderate, high. Some low shedding cows were selected for the study as they were the only MAP shedding cows on herd during the longitudinal study. MAP isolates were collected from 180 cows from 20 herds among the 376 cows analyzed by fecal culture. We failed to isolate strains in 2 herds, as when analyzing feces from these cows, non-MAP microorganisms resisted the decontamination process, showing complete overgrowth of the medium with both the original culture and subsequent repeat cultures (Whittington, 2010). The success of the culture depends essentially on the ability to control the outgrowth of other microorganisms which may vary depending on the diet of the cattle and on geographical regions (Whitlock et al., 1989; Whittington, 2009). In addition, contamination can be grouped together in samples from certain farms, which was observed for 2 farms. Cultures were performed using aliquots of frozen MAP culture preserved at −80°C, corresponding to the highest MAP shedding period (indicated by the arrow, Figure 1). The storage conditions at −80°C in absence of freeze and thaw cycles were optimal (Khare et al., 2008). Thus, we were able to collect MAP isolates from 4 to 5 years of historical samples and from low-shedding cows (Figures 1E, F). Of the 67 cattle examined further within this study, 42 were classified as high shedders (Ct < 27), 20 were classified as moderate shedders (27 < Ct < 33), and five cows were classified as low shedders (Ct > 33). Select examples of the profiles of each category of shedder are shown in Figure 1.

Genome sequencing was conducted with 67 MAP isolates that were isolated from 20 herds throughout the provinces of Quebec (QC) and Ontario (ON). These provinces are the two largest dairy producers in Canada making farms within these provinces an optimal choice for conducting a molecular epidemiology study. This study was performed in part as a proof of concept for the high-resolution mapping of MAP isolates within animals across multiple herds. By optimizing the methodology performed within this study, it will allow the validation of a pipeline to be used in the next study examining the relations of MAP at an intra-herd level.

### 3.2 Phylogenetic SNP based analysis

Of the 67 assemblies that resulted from our pipeline, an average genome size of ∼4.78 Mb, with average GC content of 69.33% and average read coverage depth of ∼14.9X (relative to K-10) was achieved (Supplementary Table S2). Verification of the assemblies using CheckM confirmed that the assemblies produced were of high quality, with an average completeness of ∼99.28% (classified as “near” completion) and average contamination of ∼1.13% (classified as “low” contamination) (Parks et al., 2015) (Supplementary Table S3).

While type II isolates are typically seen in cattle, and type I/III isolates typically found within sheep, previous studies have noted instances where these types were found in additional host organisms (Stevenson et al., 2009; Fernandez et al., 2014; Verdugo et al., 2014; Szteyn et al., 2020). While it may be expected that our isolates belong to the type II category due to their cattle-based origin, it is not guaranteed. To verify that the isolates examined in this study were type II strains, genetic variant analysis based on core SNP phylogenies for each isolate was performed using Snippy against the following MAP strains: Telford (type 1), K-10 (type II), S397 (type III), and MAPK JB-16/15 (Type B), (Supplementary Figure S3A–D, respectively). All 67 field isolates from the provinces of Quebec and Ontario were shown to be closely related to K-10 (Figure 3), with a range of 51–132 SNPs present across these 67 (Supplementary Table S4A). The genetic variant analysis performed on these 67 isolates based on the type I, III and B strains showed much higher SNP ranges of 3,333–3,429, 3,297–3,400, and 564–653, respectively (Supplementary Tables S4B–D). As expected, the lower number of variants in the K-10 based genetic variant comparison indicates that these isolates are type II strains. This claim was further validated using methods established in previously published works to examine the assemblies of each isolate and verify characteristics which match type II strains. Isolates were categorized as C-type strains through the confirmation of the C-type exclusive large sequence polymorphism (LSP) LSP^A^20 (Semret et al., 2006; Biet et al., 2012). None of the isolates contained the B-type specific “TG” deletion present in IS*1311* locus 2, confirming all isolates examined in this study as being type II strains (Sohal et al., 2010; Bryant et al., 2016).

**FIGURE 2:**
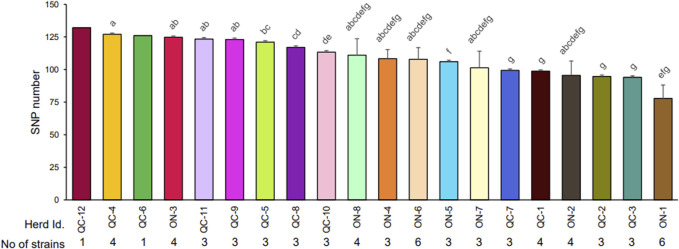
Average number of SNPs detected in the strains isolated within each herd. The number of SNPs in each strain was recorded (Supplementary Table S4A). The average number of SNPs detected in stains isolated in each herd with different letters differ significantly (*p* < 0.05) after application of a Tukey correction, as described in *Material and Methods*. The number of strains analyzed by whole genome sequencing (WGS) is reported for each herd. Each strain is derived from a single isolated colony from cow.

The total number of SNPs found varied from 51 to 132 SNPs (Supplementary Table S4A) and significantly varied among herds (Figure 2). Herds with isolates with the highest genetic variability were generally in the province of Quebec with the exception of herd ON-3. Herd prevalence of MAP infection did not correlate (r_s_ = −0.19) with the genetic variability of the isolates found in herds (Figure 2). The number of unique variants present within each assembly was also recorded (Supplementary Table S5) Of the 67 field isolates examined in this study, 13 of them contain no unique SNPs. The remaining 54 isolates all contain at least one unique SNP, with the highest number of unique SNPs being 31 in strain 1,452 (herd ON-2). When examining the number of unique SNPs within individual herds, all herds with multiple isolates were shown to have multiple isolates with their own unique MAP variant patterns.

**FIGURE 3:**
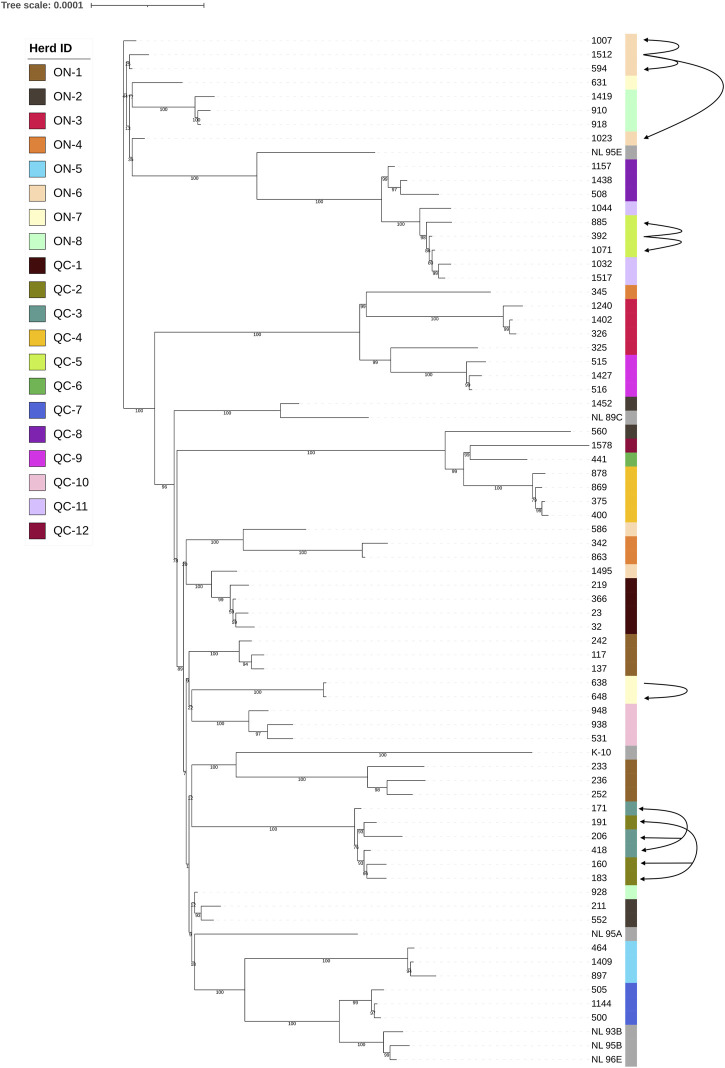
Core SNP phylogeny of Type II MAP strains. Phylogenetic tip labels are colored by herd. Tip labels in grey reflect type II strains used as reference sequences downloaded from the NCBI database. The isolates are divided into two major clades, represented by the main axis that is divided into two major branches. Arrows leading from one isolate to another are indicative of a confirmed transmission event, as described within Section 3.2. Arrows with arrowheads at both ends are present herds QC-2 and QC-3 indicate that while transfer between herds has likely occurred, the exact transmission pattern of MAP between hosts is unknown.

As shown in Figure 3, the isolates are broadly separated into two main clades. This distinction is not influenced by the province from which they originate. In most cases, isolates derived from the same herds clustered together, except for herds ON-1, ON-2, ON-4, ON-6, ON-7, and ON-8. In these herds, as shown in Figure 3, the genetic diversity (standard deviation, i.e., SNP ranges) was much higher than many of the other herds examined. Interestingly, these herds were the largest dairy farms. Three of these herds (ON-6, ON-7, and ON-8) contained isolates found in both clades present. The presence of multiple distantly related strains present within individual farms suggests separate infection events with distinct MAP isolates.

In some cases, the movements of animals between herds was known, and could be related to results found within the core SNP phylogeny (Figure 3). Herds QC-2 and QC-3 are located 5 km from each other and have been trading animals for a very long time (> 20 years). With recorded animal movements between the two long-standing dairy farms, it is not possible to trace the pattern of transmission between these two farms without having access to historical samples of early movements. Due to their frequent trade for decades, it is unsurprising that the isolates from these two independent commercial dairy farms cluster closely together within the core SNP phylogeny. Herds QC-5 and QC-11 cluster closely together as well, which can be partially explained by a recorded animal movement. The first MAP positive cows in herd QC-5 were detected in 2014. A 4 year old JD positive cow, which isolate 392 was collected from, was purchased from herd QC-11 at the age of 3 years old and started to excrete MAP on the following year. As MAP infection generally occurs in young (< 2 years old) livestock (Fecteau et al., 2010), it suggests that this cow was already infected with MAP when purchased. The introduction of this animal to herd QC-5 also coincided with the period of births of animals that were expected to become positive in 2016–2017, most notably including the cows which isolates 885 and 1,071 were collected from. This cow tracking data, along with the results observed in the core SNP tree seemingly confirm a direct ancestral transmission of MAP from herd QC-11 to QC-5.

Another potential transmission event can be found in herd ON-7. This herd contained three isolates with two distinct genetic profiles, one within isolate 631 and another within isolates 638 and 648. The animal that isolate 631 was obtained from was born within the herd in December of 2010, while the animal which isolate 638 was extracted from was born on a separate farm in October of 2010. This second cow (isolate 638) was introduced to herd ON-7 in 2012 (at age of 2 years, 2 months). The cow which isolate 648 was obtained from was born in 2013, leaving an age difference of ∼2.5 years between this cow and the cow isolate 638 was obtained from. This timeframe would allow cow 638 to transmit its MAP strain to herd mates, including cow 648, explaining the clustering observed on in Figure 3.

A third instance of potential transmission is observed in herd ON-6. This herd shows two distinct profile types, one shared by isolates 594, 1,007, 1,023, and 1,512, and another shared by isolates 1,495 and 586. The cow isolate 1,512 was derived from is the oldest of these cows, which was born in December of 2008 on a separate farm from ON-6. This cow was transferred to herd ON-6 at the age of 2 years and 4 months, while cow 594, cow 1,007 and cow 1,023 all born > 2 years later in 2010. Cow 1,512 was 5–6 years old during their birth period, allowing these three cows to become infected by cow 1,512, as suggested by their similar genetic profile. Cows that resulted in isolates 586 and 1,495 were born within herd ON-6, in 2010 and 2012 respectively. Confirmed MAP shedding for cow 586 was shedding MAP at 5 years and 6 months’ old, while confirmed MAP shedding for cow 1,495 was found at 3 years and 8 months’ old. The difference in the variant profile presented by isolates 586 and 1,495 compared to the other four isolated derived from this herd, alongside the fact that both cows were born and raised within herd ON-6, suggests that a secondary infection event was present on this herd for some time.

### 3.3 Genetics and virulence factors

As previously shown in Figure 1, the shedding of MAP within host feces can vary significantly, with some hosts shedding high amounts of MAP (Figures 1A, B) while other hosts shed MAP at a very low level (Figures 1E, F). The exact reason for this variation in shedding difference, whether it be due to the host, the bacteria, or an interaction between the two, is currently unknown (Mitchell et al., 2015). Genetic variations in the virulence factors could impact the mechanisms allowing MAP to infect the host and thwart the mechanisms of the macrophage where it nests in order to ensure its survival and multiplication. Since the information available for MAP is limited, the mode of action of the virulence factors of MAP are imputed from functions observed in related mycobacteria, such as *Mycobacterium tuberculosis* (Mtb). In addition to examining the phylogeny of SNPs and transmission patterns in herds, the presence of many SNPs in several virulence gene families may help improve our current knowledge of MAP virulence. The virulence factors explored in this study, namely the proline-glutamate/proline-proline-glutamate motif (PE/PPE) genes, mycobacterial membrane protein Large (mmpL), and the mammalian cell entry (mce) operons, were chosen on the basis of studies on the pathogenicity of Mtb and *M. avium* (Lim et al., 2021; Ssekitoleko et al., 2021). We examined the presence of genetic polymorphisms in PE/PPE, mmpL, and mce genes from each of our field isolates and noted variations present within these genes (Tables 1–3).

**TABLE 1:** Herd size, prevalence of infection per-herd and samples examined per herd for 14 herds examined within this study.

Herd no.	QC-1	QC-2	QC-3	QC-4	QC-5	QC-6	QC-7	QC-8	QC-9	QC-10	QC-11	QC-12	ON-1	ON-2	ON-3	ON-4	ON-5	ON-6	ON-7	ON-8
Herd size^a^	157	71	90	91	45	47	82	265	121	156	195	104	423	231	162	155	122	378	208	155
Prevalence (%)^b^	7.0	29.6	10.0	2.2	15.5	6.4	14.7	11.7	18.2	19.2	4.1	16.4	11.1	11.7	11.1	8.4	4.9	32.5	10.1	24.6
MAP-infected cows	11	21	9	2	7	3	12	31	22	30	8	17	47	27	18	13	6	123	21	38
Number of strains^c^	4	3	3	4	3	1	3	3	3	3	3	1	6	4	4	3	3	6	3	4

^a^ The cows were older than 24 months to be included in the study. Herd size reflects the number of cows over 24 months old when blood and faeces were collected at the first visit of the dairy farm.

^b^ Prevalence (%) represents the true prevalence of MAP-infected cows recorded during the longitudinal study of 3–5 years.

^c^ The number of strains analyzed by whole genome sequencing (WGS) is reported for each herd. The strain is derived from a single isolated colony from the selected cow.

**TABLE 2:** Mutations identified in PE/PPE proteins in reference to the MAP K-10 Genome.

PE/PPE name^a^	MAP gene/protein (K10)	TB orthologue	Location of variant^b^	Variant type^c^	Nucleotide alteration	Protein alteration	Variant effect	Herd Id^d^
MAC_PPE1	Map0123	PPE20	Between 131778–131779	INS	+CGG	Ala duplication	Disruptive Inframe Insertion	QC-2 (191), QC-3 (206), ON-2 (560)
MAC_PPE30	Map1144c	N/A	1198312	SNP	C - > G	Gln - > His	Missense mutation	QC-5, QC-8 (1,157), QC-11
MAC_PPE20	MapP1518	PPE32	1668384–1668404	DEL	-CGC​CGC​CTA​TGA​GAC​GGC​CTT	Shortening of Protein	Conservative Inframe Deletion	ON-3 (325)
MAC_PPE16	Map1676	N/A	1831523	SNP	G - > A	Gly - > Glu	Missense mutation	ON-6 (594, 1,023, 1,512), ON-7 (631), ON-8 (910, 918, 1,419)
UNK	Map1734	PPE37	Between 1896185–1896186	INS	+C	Shortening of protein	Frameshift mutation	ON-7 (638, 648)
MAC_PPE14	Map 1813c	N/A	1990165–1990219	DEL	-GCC​GCG​GCG​GCG​CCG​ACG​GGC​CCG​CGC​CTT​CTT​GCT​GCC​CGC​GGC​CGC​CGC​CGC​G	Shortening of protein	Frameshift mutation	ON-6 (594, 1,512)
MAC_PPE14	Map 1813c	N/A	1991288	SNP	G - > A	Ala - > Val	Missense mutation	QC-5, QC-8 (1,157), QC-11
MAC_PPE10	Map2575c	PPE18	2896134–2896,145	DEL	-CGCCGCCGACCG	Shortening of Protein	Disruptive Inframe Deletion	QC-10
MAC_PPE9	Map2595	N/A	2920978	DEL	-A	Shortening of protein	Frameshift mutation	All Isolates
MAC_PPE9	Map2595	N/A	2921144	SNP	C - > T	Pro - > Leu	Missense mutation	QC-2, QC-3
MAC_PPE7	Map2601	N/A	2927781	SNP	G - > A	Gly - > Arg	Missense mutation	QC-5, QC-11
MAC_PPE33	Map3185	PPE51	3536540	SNP	C - > T	Leu - > Leu	Synonymous mutation	QC-5, QC-11
MAC_PPE36	Map3490	N/A	Between 3881083 and 3881084	INS	+G	Shortening of protein	Frameshift mutation	QC-4 (400)
MAC_PPE41	Map3725	PPE3	4146287	SNP	C - > T	Asn - > Asn	Synonymous mutation	ON-6 (594, 1,023, 1,512), ON-7 (631), ON-8 (910, 918, 1,419)
MAC_PE9	Map4144	N/A	4622682	DEL	-T	Shortening of protein	Missense mutation, “intergenic region”	QC-1, QC-2 (183), QC-3 (171), QC-4 (375, 400, 869), QC-5, QC-6, QC-8 (1,157), QC-9 (516), ON-2 (211, 1,452), ON-3 (325, 326), ON-7 (638, 648)
MAC_PE9	Map4144	N/A	4622687	DEL	-T	Shortening of protein	Missense mutation, “intergenic region”	QC-1, QC-2 (183), QC-4 (375), QC-5 (392), QC-9 (515, 516), ON-3 (325, 326), ON-7 (648), QC-8 (1,157)
MAC_PE9	Map4144	N/A	4622694	DEL	-A	Shortening of protein	Missense mutation, “intergenic region”	QC-1, QC-2 (183), QC-3 (171), QC-4 (375, 869), QC-5 (392), QC-6, QC-9 (515, 516), ON-3 (325), ON-7 (648), ON-8 (1,419)

^a^ Proteins identified as described in Mackenzie et al. (2009) (https://www.ncbi.nlm.nih.gov/pmc/articles/PMC2668356/). One protein, an orthologue of M. tuberculosis protein PPE 20, was not given an annotation by this publication and is listed as unknown (UNK).

^b^ Mutations are sorted according to nucleotide locus in reference to the K-10 genome sequence (Accession ID: AE016958.1).

^c^ SNP, single nucleotide polymorphism; INS, insertion; DEL, deletion.

^d^ Listing of the herd ID, indicates that all isolates taken from that herd have the same mutation. If only certain isolates from a herd contain the mutation, they are listed in parenthesis.

**TABLE 3:** Mutations identified in mce proteins in reference to the MAP K-10 Genome.

mce gene^a^	MAP gene/protein (K10)	Location of variant (K-10)^b^	Variant type^c^	Nucleotide alteration	Protein alteration	Variant effect	Herd Id^d^
mce7-2F	Map0113	124194	SNP	C - > T	Ile - > Ile	Synonymous mutation	QC-3 (171)
mce7-2F	Map0113	124251	SNP	G - > T	Lys - > Asn	Missense mutation	QC-12
mce4F	Map0569	594004	SNP	T - > C	Leu - > Pro	Missense mutation	QC-1, QC-2, QC-3 (171), QC-7, ON-1 (117, 137, 242), ON-2 (211, 552), ON-4 (342, 863), ON-5, ON-6 (586, 1,495), ON-7 (638, 648)
mce3E	Map2112c	2338751	SNP	G - > A	Pro - > Pro	Synonymous mutation	ON-5
mce5-2E	Map2193	2436439	SNP	C - > T	Pro - > Leu	Missense mutation	QC-5, QC-8, QC-11
mce5-2E	Map2193	2436501	SNP	G - > T	Ala - > Ser	Missense mutation	QC-1, QC-2 (183, 191), QC-3 (206, 418), QC-4 (375, 400, 869), QC-5 (885, 1,071), QC-7, QC-8 (1,157, 1,438), QC-9, QC-10 (938, 948), ON-1 (117, 233, 236, 252), ON-2 (211, 552, 560), ON-3 (326, 1,240), ON-4 (342), ON-5, ON-6 (594, 1,495, 1,512), ON-7 (631), ON-8
mce5-2F	Map2194	2437285	SNP	G - > C	Val - > Leu	Missense mutation	ON-1 (117, 137 and 242)
mce5-2F	Map2194	2437484	SNP	C - > G	Ala - > Gly	Missense mutation	All strains
Independent mce	Map3289c	3652375	SNP	G - > C	Pro - > Pro	Synonymous mutation	QC-6
Independent mce	Map3289c	3652383	SNP	T - > G	Ile - > Leu	Missense mutation	QC-6
Independent mce	Map3289c	3653470	SNP	C - > G	Thr - > Thr	Synonymous mutation	QC-6
yrbE1B	Map3603	3999143	DEL	-G	Extension of protein	Frameshift mutation	All Isolates
mce1A	Map3604	4000027	SNP	C - > G	Thr - > Thr	Synonymous mutation	QC-10 (938)
mce1A	Map3604	4001149	SNP	G - > C	Pro - > Pro	Synonymous mutation	QC-5, QC-8, QC-11
mce1F	Map3609	4006938	SNP	C - > G	His - > Gln	Missense mutation	QC-5 (885)
yrbE2B	Map4083	4553018	SNP	T - > C	Tyr - > His	Missense mutation	QC-5, QC-8, QC-11
yrbE2B	Map4083	4553276–4553278	DEL	-GTG	-Val, Shortening of protein	Disruptive Inframe Deletion	QC-9, ON-3 (326, 1,240), ON-4 (345)
yrbE2B/mce2A	Map4083/Map4084	4553474	DEL	-C	Extension of protein mce2A	Frameshift mutation	All isolates
mce2D	Map4087	4557531	SNP	T - > C	Val - > Ala	Missense mutation	All isolates
Independent mce	Map3289c	3653477–3653478	Complex	GC - > TT	Ala - > Asn	Missense mutation	QC-6
mce1A	Map3604	4000019–4000020	MNP	AA - > GC	Asn - > Ala	Missense mutation	QC-10 (938)

^a^ Proteins identified as described in Hemati et al. (2019) (https://pubmed.ncbi.nlm.nih.gov/31282842/).

^b^ Mutations are sorted according to nucleotide locus in reference to the K-10 genome sequence (Accession ID: AE016958.1).

^c^ SNP, single nucleotide polymorphism; INS, insertion; DEL, deletion, Complex = Combination of SNP/multi-nucleotide polymorphism.

^d^ Listing of the herd ID, indicates that all isolates taken from that herd have the same mutation. If only certain isolates from a herd contain the mutation, they are listed in parenthesis.

PE and PPE protein families are proteins unique to mycobacteria and are suspected to have roles in the pathogenicity of the organism (Sampson, 2011; Timms et al., 2015). These proteins are typically found at the cell surface, with conserved N-terminal and C-terminal domains that broadly categorize these proteins into subfamilies (Pal et al., 2019). Some PE/PPE proteins have been shown to elicit B cell responses (Sampson, 2011) and affect macrophage or dentritic cells function (Brennan et al., 2001; Bansal et al., 2010). With some PE/PPE proteins expressed on the cell surface, one would expect to have a differential antigenic response or pathogenetic variation associated with the different genetic patterns of these genes. The WGS analysis reveals several missense, frameshift, and synonymous mutations, and also genetic variations inducing a disruptive inframe deletion in several PE/PPE genes (Table 2). Genetic variation was found in only one PE gene, in locus MAC_P9 (Map4144 protein). In several isolates, the deletions (missense mutations) identified would truncate the Map4144 protein. Interestingly, virulence and antigenicity of MAP during macrophage infection are dominated by the up-regulation of Map4144 (Cossu et al., 2012). This gene family showed a high degree of conservation (Sampson, 2011). Whether the missense mutation affect Map4144 function worth future investigation. In PPE, genetic variations were detected in 12 PPE genes, notably in locus MAC_PPE1, −7, −9, −10, −14, −16, −20, −30, −33, −36, and MAC_PPE41 (Table 2). All of them are conserved in other MAC bacteria with the exception of 7 of these proteins having no orthologue in Mtb (Mackenzie et al., 2009). The literature does not report a functional role for them either. The remaining six locus, corresponding to Map0123 (MAC_PPE1), Map2575c (MAC_PPE10), Map1518 (MAC_PPE20), Map3185 (MAC_PPE33), Map3725 (MAC_PPE41) and Map1734 (uncharacterized MAP locus) proteins, have orthologues in Mtb, corresponding to PPE20, PPE18, PPE32, PPE51, PPE3, and PPE37, respectively. Given the importance of the PE/PPE family for virulence, and mutations that could affect the host antigenic response, PE/PPE identified with a mutation affecting their functions should be further investigated.

Factors that enable the pathogen to achieve infection and allowing persistence are also considered to be virulence factors. Genetic variations in mammalian cell entry (mce) operons, implicated in the invasion of mycobacteria into host cells, are expected to influence MAP virulence as well. MAP contains eight *mce* operons instead of the four present in Mtb, with two copies of the *mce5* and *mce7* operons (Casali and Riley, 2007; Hemati et al., 2019). The structure of *mce* genes in each operon is composed of eight genes (*mceA–mceF* and *yrbEA-B*). Several missense mutations were identified in *mce1A*, *mce1F*, *mce5-2E*, *mce5-2F*, *mce7-2F* and *yrbE1B* and *yrbE2B* genes (Table 3).

Cell envelope is a critical aspect of mycobacterial physiology and virulence and mycobacterial membrane protein large (mmpL) are permeases which are an integral part of their membrane (Melly and Purdy, 2019). These membrane transporters indirectly contribute to virulence in Mtb *via* the transport of substrates that directly influence bacterial survival. While information regarding the function these mmpL proteins is more detailed for Mtb (Melly and Purdy, 2019), their role in MAP is poorly understood (Lim et al., 2021) and will deserve future investigation.

### 3.4 *In-silico* analysis of repetitive elements

The variable-number tandem repeats (VNTRs) like the mycobacterial interspersed repetitive units (MIRU) or the multilocus short sequence repeat (SSR) are a significant source of genetic polymorphisms in mycobacteria, including MAP. With low mutation rate of this slow-growing organisms, the DNA repetitive elements are one of the main driving forces of their genome evolution. Multi locus variant analysis (MLVA) provides a discriminatory power and more importantly, the portability of the results (MLVA codes) facilitates their exchange between laboratories. *In-silico* analysis of eight MIRU-VNTR loci, namely MIRUs 292 and X3 and VNTRs 25, 47, 3, 7, 10 and 32 (Thibault et al., 2007), and 11 SSR loci (Amonsin et al., 2004) was performed on the 67 isolates from the 20 dairy herds and the NCBI strain references, notably K-10, MAPK JB16/15 (B type strain), Telford (Type I strain), and the six isolates from the province of Newfoundland and Labrador (NL), Canada (Podder et al., 2015). The repeat size at each locus was recorded manually for each isolate, along with the herd Id, province Id, and MAP shedding level of the 67 cows from which they were isolated (Supplementary Table S1).

**FIGURE 4:**
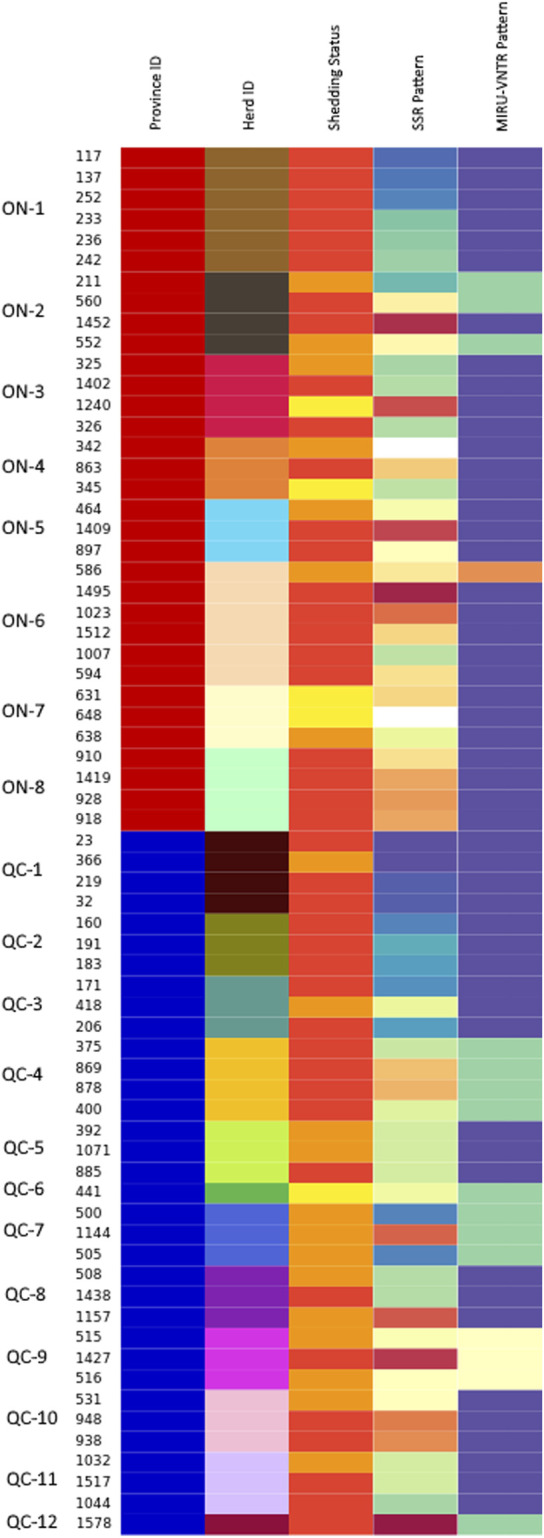
Visualization of the distribution of shedding status, SSR type and MIRU-VNTR type across different herds in ON and QC. Isolates are grouped by herd, with colors in each column reflecting a different value within their respective category. In the province column, isolates from QC are labeled in blue, while isolates from ON are labeled in red. Herd colors are the same as those in Figures 2, 3 and are labelled along the side of the figure. The shedding status column identifies isolates from animals that were high shedders (red), moderate shedders (orange) and low shedders (yellow). Isolates with no shedding data were colored grey. Both the SSR type and MIRU-VNTR type columns use colors to represent patterns, with unknown patterns colored white.

Among the 67 isolates examined with SSR markers, loci 1 and 2 showed the highest discriminatory index (DI) values at 0.8508 and 0.8005, respectively (Supplementary Table S1). For SSR locus 1, a total of 15 distinct repeat patterns were identified, while SSR locus 2 had a total of eight distinct repeat patterns (Supplementary Table S1). Other SSR loci had only one repeat pattern, including SSR’s 3, 4, 5, 10 and 11, thus excluding their discriminatory capability. Apart from the high discriminatory power of loci 1 and 2, SSR loci 7 (DI of 0.4242) displayed 3 different patterns, followed by loci 6, 8, and 9 with a much lower DI (0.0299 each), with only 2 patterns. Among the MIRU-VNTR loci examined, locus 10 was the most discriminatory locus (DI of 0.2985), followed by locus 292 (DI of 0.0882) and locus 7 (0.0298), all with only 2 different repeat patterns present within the 67 field isolates examined. The remaining MIRU-VNTR loci were not informative. All MIRU-VNTR *in silico* results matched those obtained by PCR (data not shown). As shown in Figure 4, multilocus SSR markers were more informative than MIRU markers. The DIs from the MIRU-VNTR markers were globally lower than the DIs from the SSR markers, which has been reported in other studies (Ahlstrom et al., 2014; de Kruijf et al., 2017).

Examination of the MIRU-VNTR and SSR loci was also performed using the web application called “MAC-INMV-SSR database, a database which contains the MIRU-VNTR and SSR profiles of several known strains of MAP (Cochard et al., 2020). Using this database to examine the 67 Type II isolates from the provinces of Quebec (QC) and Ontario (ON), we identified 4 different INMV-MIRU patterns (Supplementary Table S5). The predominant type of the field isolates in these provinces was INMV 2 (76.1%), followed by INMV 3 (17.9%) and INMV 13 (4.5%). The group INMV eight recorded only on isolate. The DI using the INMV database patterns is 0.3740.

Analysis of the INMV-SSR pattern was obtained for 67 isolates from QC and ON. Some strains (8) had an ambiguous number of repeats in their assembly (for the field isolates assembled) or a lack of coverage over the entire repeat (for the reference sequences downloaded from NCBI). Manual examination of the repeats allowed for the confirmation of the SSR patterns of six of the field isolate assemblies, leaving the remaining 2 field isolates, along with 2 NCBI sequences, listed as having an unknown pattern. Of the remaining 74 isolates, 15 recognized patterns were detected, along with 16 novel patterns which were not present within the MAC-INMV-SSR database. An important point of consideration is that the MAC-INMV database has a slight limitation when examining SSR loci. If the repeat value is greater than 11, typical within the longer mononucleotide repeats in SSR loci 1 and 2, then the repeat value is called as “+”. This results in a slight underestimation of the true diversity of the population. Notwithstanding, the stability of the SSR locus 2 is affected by *in vivo* passage (Kasnitz et al., 2013), which may not be suitable for epidemiological study. Four of the patterns within the database (SSR 7, SSR 8, SSR 10, and SSR 93) which called long repeats as “+” were found to have several repeat “sub-patterns” within each called pattern (2 in SSR 7, 2 in SSR 8, 6 in SSR 10 and 5 in SSR 93), showing further diversity within listed repeat patterns. Among the 16 novel patterns, 6 were found to have additional patterns that would not be recognized due to their high repeat values. When taking these additional patterns into account, the DI value increases from 0.9457 to 0.9846, which is reported in Table 4 and Supplementary Table S1.

**TABLE 4:** MLSSR and MIRU-VNTR Patterns according to the MAC-INMV database.

Strain Id^a^	Herd Id^b^	Province	Shedding status	INMV SSR type^c^	INMV MIRU type^c^
23	QC-1	QC	High	MLSSR 10a	INMV 2
32	QC-1	QC	High	MLSSR 38	INMV 2
117	ON-1	ON	High	N/P 12a	INMV 2
137	ON-1	ON	High	N/P 15	INMV 2
160	QC-2	QC	High	MLSSR 10d	INMV 2
171	QC-3	QC	High	MLSSR 10c	INMV 2
183	QC-2	QC	High	MLSSR 8a	INMV 2
191	QC-2	QC	High	MLSSR 10b	INMV 2
206	QC-3	QC	High	MLSSR 8a	INMV 2
211	ON-2	ON	Moderate	MLSSR 8b	INMV 3
219	QC-1	QC	High	MLSSR 38	INMV 2
233	ON-1	ON	High	MLSSR 93e	INMV 2
236	ON-1	ON	High	MLSSR 93b	INMV 2
242	ON-1	ON	High	N/P 12b	INMV 2
252	ON-1	ON	High	MLSSR 10d	INMV 2
325	ON-3	ON	Moderate	MLSSR 17	INMV 2
326	ON-3	ON	High	MLSSR 11	INMV 2
342	ON-4	ON	Moderate	UNK	INMV 2
345	ON-4	ON	Low	N/P 3a	INMV 2
366	QC-1	QC	Moderate	MLSSR 10a	INMV 2
375	QC-4	QC	High	N/P 14	INMV 3
392	QC-5	QC	Medium	MLSSR 13	INMV 2
400	QC-4	QC	High	N/P 10b	INMV 3
418	QC-3	QC	Moderate	MLSSR 10e	INMV 2
441	QC-6	QC	Low	N/P 11a	INMV 3
464	ON-5	ON	Moderate	MLSSR 93c	INMV 2
500	QC-7	QC	Moderate	MLSSR 10d	INMV 3
505	QC-7	QC	Moderate	MLSSR 10d	INMV 3
508	QC-8	QC	Moderate	MLSSR 11	INMV 2
515	QC-9	QC	Moderate	MLSSR 16	INMV 13
516	QC-9	QC	Moderate	N/P 6a	INMV 13
531	QC-10	QC	Moderate	MLSSR 10f	INMV 2
552	ON-2	ON	Moderate	MLSSR 7a	INMV 3
560	ON-2	ON	High	N/P 8	INMV 3
586	ON-6	ON	Moderate	MLSSR 93a	INMV 8
594	ON-6	ON	High	N/P 4a	INMV 2
631	ON-7	ON	Low	N/P 1	INMV 2
638	ON-7	ON	Moderate	MLSSR 10e	INMV 2
648	ON-7	ON	Low	UNK	INMV 2
863	ON-4	ON	High	N/P 6b	INMV 2
869	QC-4	QC	High	N/P 10a	INMV 3
878	QC-4	QC	High	N/P 13	INMV 3
885	QC-5	QC	High	MLSSR 13	INMV 2
897	ON-5	ON	High	MLSSR 10f	INMV 2
910	ON-8	ON	High	N/P 4a	INMV 2
918	ON-8	ON	High	N/P 2	INMV 2
928	ON-8	ON	High	N/P 16	INMV 2
938	QC-10	QC	High	MLSSR 93d	INMV 2
948	QC-10	QC	High	N/P 9b	INMV 2
1,007	ON-6	ON	High	N/P 3a	INMV 2
1,023	ON-6	ON	High	N/P 4b	INMV 2
1,032	QC-11	QC	Moderate	MLSSR 13	INMV 2
1,044	QC-11	QC	High	MLSSR 17	INMV 2
1,071	QC-5	QC	Moderate	MLSSR 13	INMV 2
1,140	QC-7	QC	Moderate	MLSSR 7b	INMV 3
1,157	QC-8	QC	Moderate	N/P 3b	INMV 2
1,240	ON-3	ON	Low	N/P 3c	INMV 2
1,402	ON-3	ON	High	MLSSR 11	INMV 2
1,409	ON-5	ON	High	N/P 9a	INMV 2
1,419	ON-8	ON	High	N/P 2	INMV 2
1,427	QC-9	QC	High	MLSSR 58	INMV 13
1,438	QC-8	QC	High	MLSSR 11	INMV 2
1,452	ON-2	ON	High	MLSSR 28	INMV 2
1,495	ON-6	ON	High	N/P 5	INMV 2
1,512	ON-6	ON	High	N/P 1	INMV 2
1,517	QC-11	QC	High	MLSSR 13	INMV 2
1,578	QC-12	QC	High	N/P 7	INMV 3
K-10	NCBI	N/A	N/A	MLSSR 10d	INMV 3
NL-89C	NCBI	NL	N/A	MLSSR 3	INMV 68
NL-93B	NCBI	NL	N/A	MLSSR 54	UNK
NL-95A	NCBI	NL	N/A	MLSSR 52	UNK
NL-95B	NCBI	NL	N/A	MLSSR 17	N/P 1
NL-95E	NCBI	NL	N/A	MLSSR 17	UNK
NL-96E	NCBI	NL	N/A	MLSSR 38	UNK
MAPK_JB16/15	NCBI	N/A	N/A	MLSSR 29	UNK
S397	NCBI	N/A	N/A	UNK	UNK
Telford	NCBI	N/A	N/A	UNK	INMV 72
Discriminatory Index				0.9846	0.3740

^a^ Typing of isolates from Newfoundland Canada (NL-89C/93B/95A/95B/96E) were published (http://www.ncbi.nlm.nih.gov/pubmed/25927612).

^b^ Values from NCBI, database are not included in DI, calculations.

^c^ N/P = New Patterns. Patterns not recognized as a type in the INMV, database. Patterns with the same number but different letters would be classified as the same type in the INMV, database but have different repeat values; UNK, Unknown. These patterns can’t be predicted due to a lack of certainty within specific loci and were not included in DI, calculations.

The INMV-MIRU-VNTR DI value of the 67 isolates was evaluated at 0.3740 while the INMV-SSR was 0.9846. The capacity of INMV-SSR to differentiate MAP isolates exceeds that established using the alternative method previously reported (DI of 0.795), named the large gaps and tandem repeats factors (Lim et al., 2021), making of the web application “MAC-INMV-SSR database” the best reference for reporting typed MAP.

Comparison of both multilocus SSR and MIRU-VNTR patterns, as shown in both Table 5; Figure 4, allows for the clear visualization of how these patterns are spread throughout the isolates examined. Among the 18 herds with multiple isolates, MIRU-VNTR was only able to identify multiple patterns in herds ON-2 and ON-6, each confirmed as having two patterns. All other herds contained a single INMV type each. Using SSR loci, multiple patterns were confirmed in 17 of these 18 herds, with a minimum of 2 confirmed profiles (7 herds) and a maximum of 6 confirmed profiles (ON-6). A single pattern (SSR locus 13) was confirmed in the herd QC-9 (Id 1,427). While the isolates were shown to be closely related by core-SNP analysis using ∼ 107 SNP, each isolate was confirmed to have their own pattern of variants, with no clonal isolates identified. The differentiation provided by both core-SNP analysis and MLSSR typing supports previous work which has noted that repetitive elements, especially MIRU-VNTR, are often subject to homoplasy (Ahlstrom et al., 2015; Bryant et al., 2016). In these cases, while the MIRU-VNTR pattern may be the same, further examination would reveal that the isolates are genetically distinct from one another and are unrelated. Homoplasy was obvious within the isolates examined in our study, as the broad classification of unrelated isolates as INMV 2 indicates. Homoplasy within INMV 2, itself a commonly identified MIRU-VNTR type in both Europe and Canada (Stevenson et al., 2009; Ahlstrom et al., 2015; Perets et al., 2022), was also noted as being an example of homoplasy in prior works (Ahlstrom et al., 2015; Bryant et al., 2016).

**TABLE 5:** Mutations identified in mmpL proteins in reference to the MAP K-10 Genome.

MAP gene/protein (K10)^a^	Location of variant (K-10)^b^	Variant type^c^	Nucleotide alteration	Protein alteration	Variant effect	Herd id (Isolate)^d^
Map1738	1899360	SNP	C - > T	Ala - > Val	Missense mutation	ON-6 (594, 1,023, 1,512)
Map2232	2494145	SNP	C - > T	Ala - > Ala	Synonymous mutation	QC-4, QC-6, QC-12, ON-2 (560)
Map2239	2502885	SNP	A - > G	Asp - > Gly	Missense mutation	QC-2, QC-3
Map2324c	2606590	SNP	A - > T	Leu - > Gln	Missense mutation	ON-8 (910, 918, 1,419)
Map2324c	2606598	SNP	G - > A	Thr - > Thr	Synonymous mutation	QC-4, QC-6, QC-12, ON-2 (560)
Map2324c	2607271	SNP	G - > A	Ala - > Val	Missense mutation	QC-9, ON-3, ON-4 (345)
Map2324c	2607913	SNP	T - > C	Asn - > Ser	Missense mutation	ON-6 (594, 1,023, 1,512), ON-7 (631), ON-8 (910, 918, 1,419)
Map3049c	3393600	SNP	G - > A	Pro - > Ser	Missense mutation	QC-1, ON-6 (1,495)
Map3751	4187170	SNP	A - > G	Asn - > Asp	Missense mutation	QC-10
Map3751	4188170	DEL	-A	Shortening of protein, conversion of initial gene into two proteins annotated as “MMPL family transporter CDS”	Frameshift mutation	ON-1 (117, 137, 242)
Map3890	4354347	SNP	G - > A	Asp - > Asn	Missense mutation	ON-7 (631)

^a^ Proteins identified as described in Marri et al. (2006) (https://pubmed.ncbi.nlm.nih.gov/17064286/).

^b^ Mutations are sorted according to nucleotide locus in reference to the K-10 genome sequence (Accession ID: AE016958.1).

^c^ SNP, single nucleotide polymorphism; INS, insertion; DEL, deletion, Complex = Combination of SNP/multi-nucleotide polymorphism.

^d^ Listing of the herd ID, indicates that all isolates taken from that herd have the same mutation. If only certain isolates from a herd contain the mutation, they are listed in parenthesis.

## 4 Conclusion

In summary we genetically characterized MAP isolates from 67 cattle from 20 herds within the provinces Quebec and Ontario, using different typing methods including the WGS analysis. The assemblies produced using our prospective pipeline were verified as being of high quality without contamination, which avoids the generation of misleading hypothesis. All isolates were type II, the cattle type. They were subtyped using both traditional MLVA typing methods such as MIRU-VNTR and MLSSR. Examination of the SSR and MIRU-VNTR based methods showed that SSR had significantly more discriminatory power than MIRU-VNTR, as it was able to identify more unique patterns both across the entire dataset and within each of the individual herds. However, the limited discriminatory capacity of the multilocus typing methods does not allow tracking inter-herd MAP transmission. The core SNP-based analysis was the only approach leading to assign a individual signature to each isolate and to document disease transmission across herds, confirmed by animal movement data. While the structure of the 67 isolates within the phylogenetic tree was not correlated with the phenotypes recorded in the animals, the presence of genetic variations in several virulence genes, notably for PE, PPE, mce and mmpL, could explain differential antigenic or pathogenetic responses. Additional isolates are being studied and the analysis of a larger population should make it possible to study the impact of the genetic pattern of MAP virulence genes and their impact on the host in a context of controlled infection.

## Data Availability

The 67 datasets presented in this study are associated with the BioProject PRJNA925907 and can be found in online repositories under the accession numbers: SRR23179790 to SRR23179856.
