# Supplementary material for: Genomic epidemiology of Mycobacterium avium subsp. paratuberculosis isolates from Canadian dairy herds provides evidence for multiple infection events
Source: Front Genet. 2023 Feb 2;14:1043598. doi: 10.3389/fgene.2023.1043598 (PMC9934062; doi:10.3389/fgene.2023.1043598)
Supplement: Supplementary file 1 [file Image1.pdf]

Supplementary Material

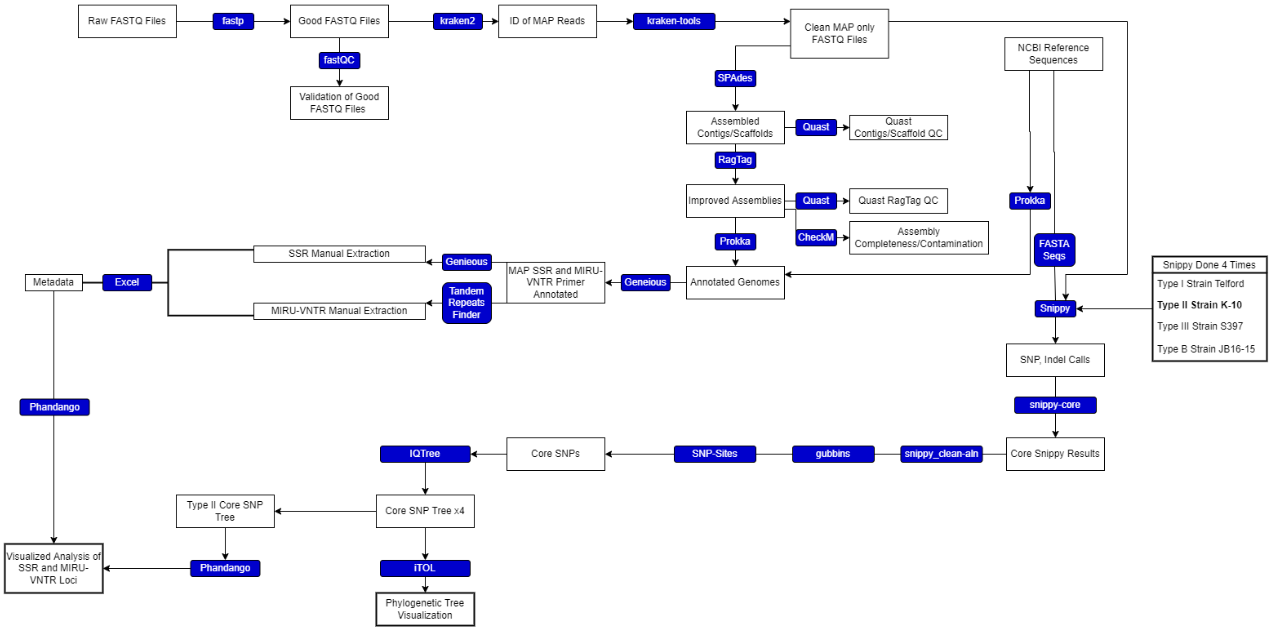

Supplementary Figure S1: Pipeline of bioinformatic tools (blue) and files (white) used to analyze whole genome sequencing data. Exact parameters used for each tool are described in the main manuscript.

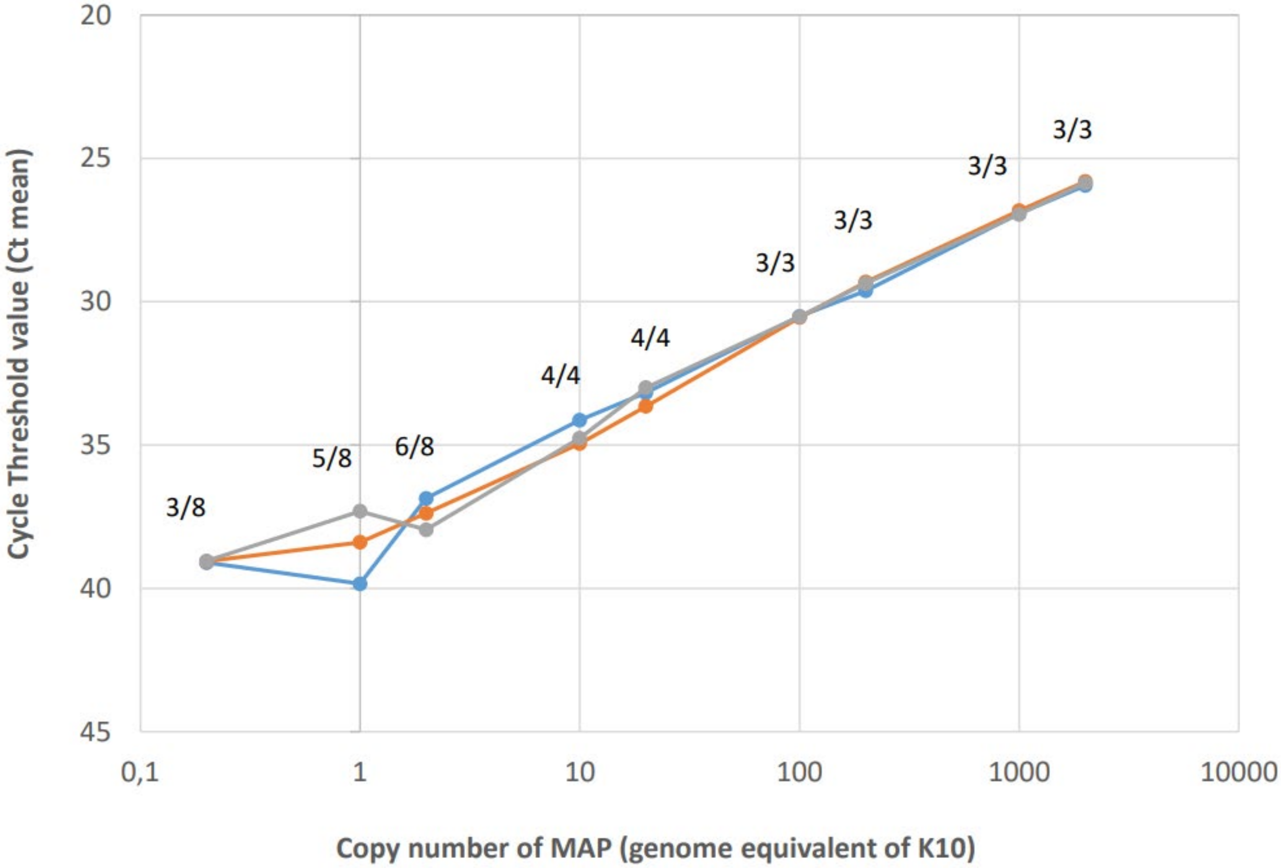

Supplementary Figure S2: Detection of K10 strain (ATCC BAA-968D-5) of Mycobacterium avium subsp. paratuberculosis (MAP) by quantitative PCR (qPCR). Serial dilutions of DNA corresponding to known quantities of genome equivalents (Ge) were quantified by qPCR. Three independent serial dilution curves were made. Each dilution point was quantified eight times for the dilutions of 0.2, 1 and 2 Ge, four times for 10 and 20 Ge, and three times for the dilutions of 100, 200, 1000 and 2000 Ge.

A)

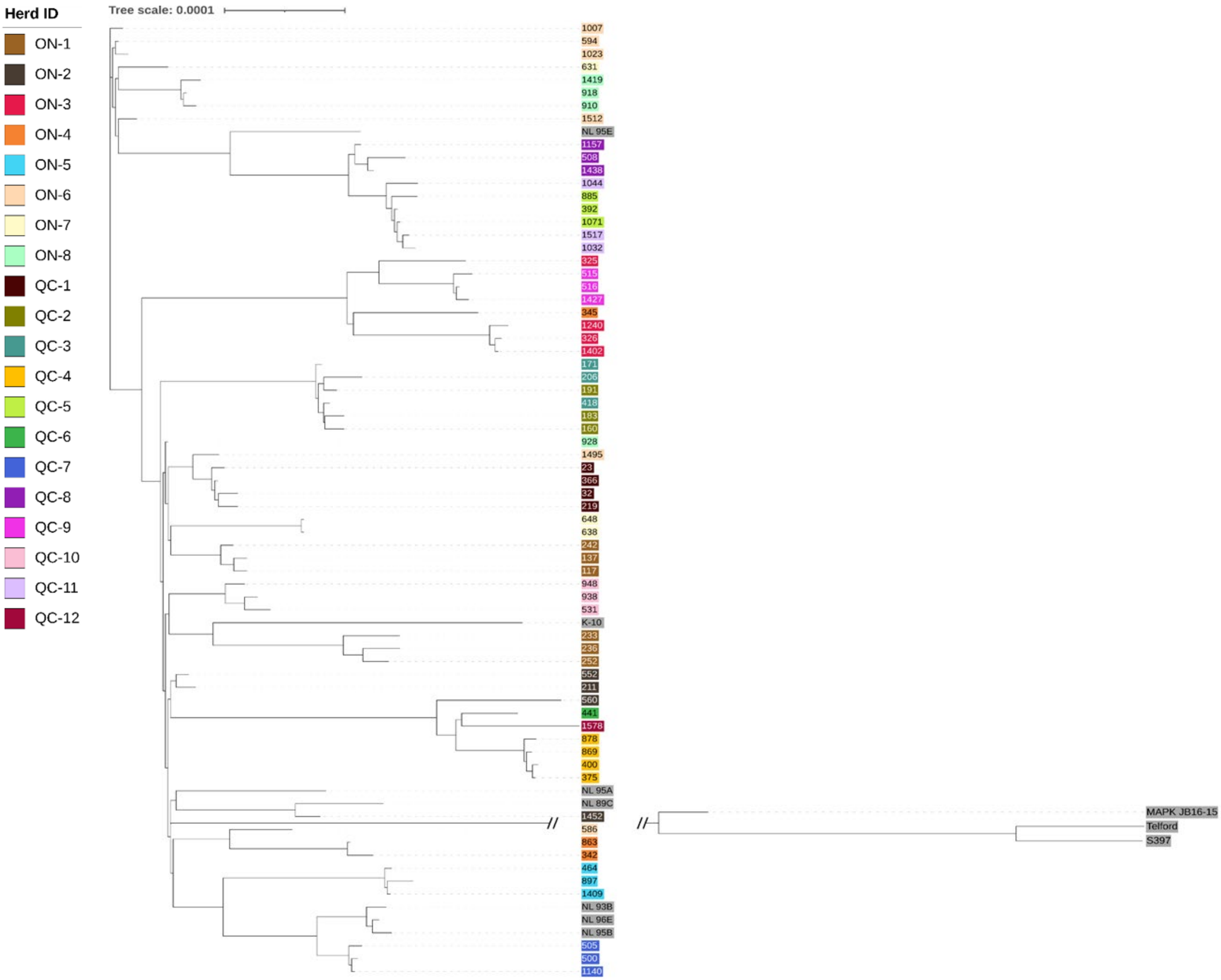

Herd ID
ON-1
ON-2
ON-3
ON-4
ON-5
ON-6
ON-7
ON-8
QC-1
QC-2
QC-3
QC-4
QC-5
QC-6
QC-7
QC-8
QC-9
QC-10
QC-11
QC-12
Tree scale: 0.0001
1007
594
1023
631
1419
918
910
1512
NL 95E
1157
508
1438
1044
885
392
1071
1517
1032
325
515
516
1427
345
1240
326
1402
171
206
191
418
183
160
928
1495
23
366
32
219
648
638
242
137
117
948
938
531
K-10
233
236
252
552
211
560
441
1578
878
869
400
375
NL 95A
NL 89C
1452
586
863
342
464
897
1409
NL 93B
NL 96E
NL 95B
505
500
1140
MAPK JB16-15
Telford
S397

B)

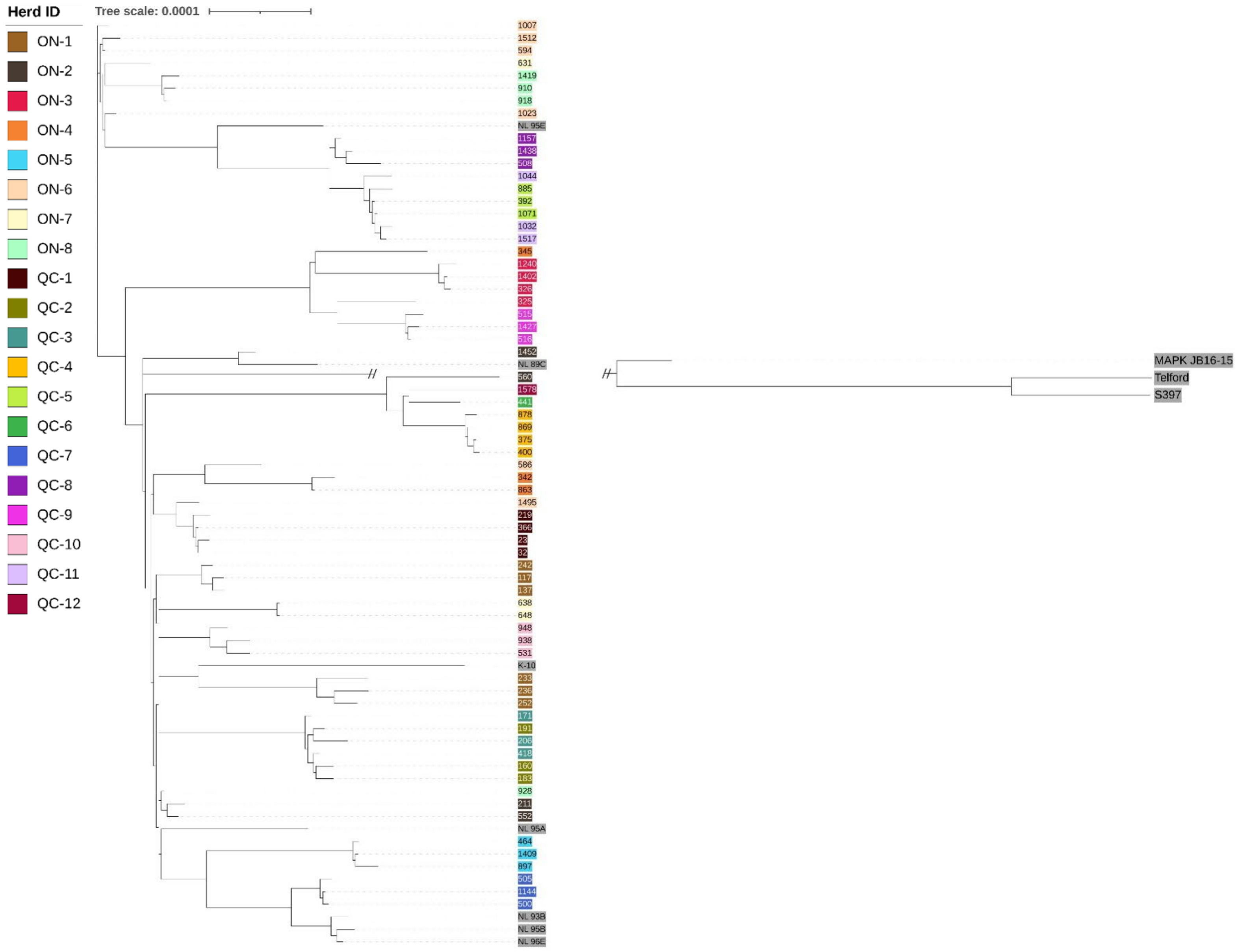
Herd ID
ON-1
ON-2
ON-3
ON-4
ON-5
ON-6
ON-7
ON-8
QC-1
QC-2
QC-3
QC-4
QC-5
QC-6
QC-7
QC-8
QC-9
QC-10
QC-11
QC-12
Tree scale: 0.0001
MAPK JB16-15
Telford
S397

C)

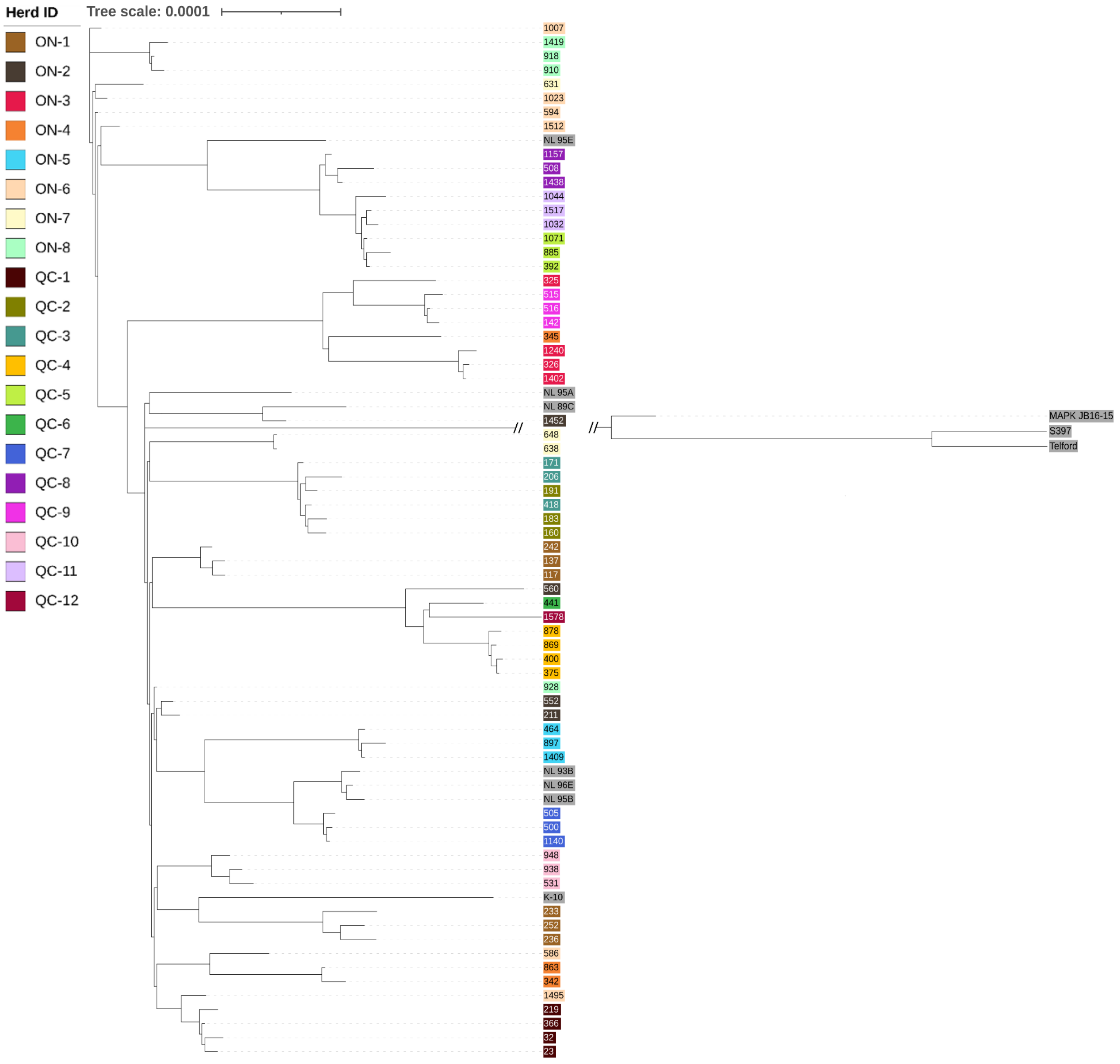
Herd ID
ON-1
ON-2
ON-3
ON-4
ON-5
ON-6
ON-7
ON-8
QC-1
QC-2
QC-3
QC-4
QC-5
QC-6
QC-7
QC-8
QC-9
QC-10
QC-11
QC-12
Tree scale: 0.0001
1007
1419
918
910
631
1023
594
1512
NL 95E
1157
508
1438
1044
1517
1032
1071
885
392
325
515
516
142
345
1240
326
1402
NL 95A
NL 89C
1452
648
638
171
206
191
418
183
160
242
137
117
560
441
1578
878
869
400
375
928
552
211
464
897
1409
NL 93B
NL 96E
NL 95B
505
500
1140
948
938
531
K-10
233
252
236
586
863
342
1495
219
366
32
23
MAPK JB16-15
S397
Telford

D)

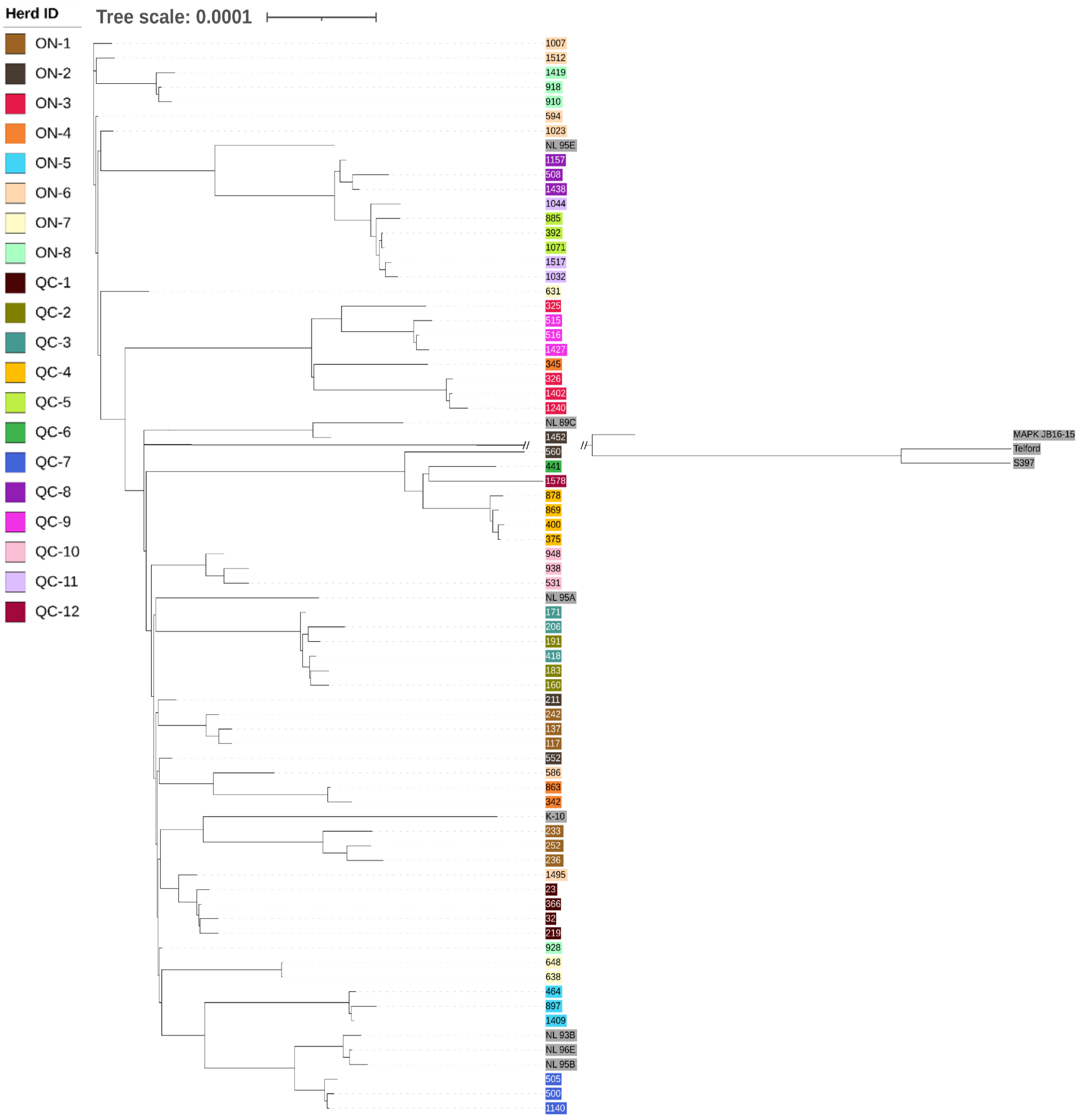

Supplementary Figure S3: Core SNP phylogenies of MAP isolates (n=67) from ON and QC herds along with select reference sequences from NCBI (n=10). Each phylogeny was constructed based on

four separate core SNP analysis, each done using a different reference strain. A) used the type I reference strain Telford (NZ_CP033688.1), B) used the type II reference strain K-10 (NC_002944.2), C) used the type III reference strain S397 (NZ_CP053749.1), and D) used the type B reference strain MAPK_JB16/15 (NZ_CP033911.1). Each of 67 field isolates are colored by herd according to the listed key, while NCBI reference sequences are all colored in grey.
